# Associations between sleep quality and well-being among nurses: the serial mediating roles of psychological resilience and adversity coping

**DOI:** 10.3389/fpsyg.2026.1962283

**Published:** 2026-09-30

**Authors:** Yan Cai, Jiayu Mao, Chunqing Yang, Ping Gao, Hanlin Ji, Yunna Zhao, Hao Chen, Junzhi Xiang, Xiaoqun Xu

**Affiliations:** 1 The First School of Medicine (School of Information and Engineering), Wenzhou Medical University, Wenzhou, China; 2 School of Nursing, Wenzhou Medical University, Wenzhou, China; 3 Department of Wound Repair, The First Affiliated Hospital, Wenzhou Medical University, Wenzhou, China; 4 Department of Emergency Medicine, The First Affiliated Hospital, Wenzhou Medical University, Wenzhou, China; 5 Department of Nursing, The First Affiliated Hospital, Wenzhou Medical University, Wenzhou, China

**Keywords:** adversity quotient, chain mediating effect, nurse, psychological resilience, sleep quality, well-being

## Abstract

**Background:**

Promoting nurses’ well-being is important for mental health and workforce stability. Poor sleep quality is common among nurses and has been associated with lower general well-being. Psychological resilience and adversity quotient may serve as protective personal resources, but their roles in this association remain unclear.

**Objectives:**

This study examined the association between sleep quality and general well-being among Chinese nurses from the perspective of conservation of resources theory, focusing on the independent and serial mediation of psychological resilience and adversity quotient.

**Methods:**

This multicenter cross-sectional study enrolled 1,149 nurses from 22 tertiary medical institutions in Zhejiang Province, China, between September and November 2025. Data were collected using the Pittsburgh Sleep Quality Index, the Adversity Quotient Scale, the Connor-Davidson Resilience Scale, and the General Well-being Schedule. Descriptive statistics, Pearson correlation analysis, and common method bias tests were conducted for the study variables. Confirmatory factor analysis and structural equation modeling were used to evaluate the measurement and hypothesized structural models, respectively. Indirect effects were tested using bias-corrected bootstrapping with 5,000 resamples.

**Results:**

Sleep quality, psychological resilience, adversity quotient, and general well-being were all significantly correlated (*P* < 0.001). Psychological resilience and adversity quotient significantly mediated the association between sleep quality and general well-being. The total indirect effect accounted for 41.6% of the total effect. All three indirect effects were significant: (1) sleep quality → psychological resilience → general well-being (β = −0.137, *P* < 0.001, 22.2% of the total effect), (2) sleep quality → adversity quotient → general well-being (β = −0.070, *P* < 0.001, 11.2% of the total effect), and (3) sleep quality → psychological resilience → adversity quotient → general well-being (β = −0.051, *P* < 0.001, 8.2% of the total effect).

**Conclusion:**

Sleep quality was associated with Chinese nurses’ well-being both directly and indirectly through psychological resilience, adversity quotient, and their serial mediation effect. Nursing managers may consider sleep quality improvement and targeted strategies to strengthen psychological resilience and adversity quotient as potential approaches to supporting nurses’ general well-being.

## Introduction

With the continuous challenges posed by recurrent COVID-19 cases and novel infectious diseases such as monkeypox, global concerns about the long-term public health consequences of infectious outbreaks remain intense (Organization, 2024; Jadhav et al., 2025). These public health emergencies can place substantial psychological and physical demands on frontline clinical nurses (Wang et al., 2023; Bai et al., 2025). As a cornerstone of healthcare delivery, nurses play a pivotal role in safeguarding patient safety and improving the quality of nursing care (Samur and Seren Intepeler, 2019). However, amidst these ongoing challenges, nursing professionals have been subjected to substantial occupational strain and demanding workloads, particularly within healthcare settings in China. While the total number of nurses in China exceeded 6 million by the end of 2025, the nurse-to-physician ratio stands at only 1:1.15, remaining significantly below the internationally recognized benchmark of 1:2 (Fu et al., 2025). This imbalance is associated with diminished general well-being (Yu et al., 2019). General well-being reflects individuals’ life satisfaction and serves as an important psychological resource for nurses and a key determinant of workforce stability and clinical practice (Bartels and Boomsma, 2009; Chen et al., 2024). However, nurses report a low-to-moderate level of well-being, which is notably lower than that of other occupational groups (Csima et al., 2024). Therefore, there is an urgent need to identify the influencing factors and pathways of their well-being, thereby providing empirical evidence to improve nursing professionals’ well-being.

Existing evidence suggests that sleep quality is closely associated with nurses’ mental health, particularly general well-being (Norful et al., 2022; Sella et al., 2023). As a vital restorative process, good sleep quality is essential not only for physical recovery but also for maintaining daily occupational functioning and emotional stability (Cecere et al., 2023). However, the underlying relational pathways through which sleep quality is associated with general well-being remain insufficiently explored. Sleep disturbances are highly prevalent among nursing professionals worldwide, with more than half experiencing poor sleep quality (Zeng et al., 2020; Gu et al., 2024). Sleep quality may serve as an important process for psychological resource restoration (Asadullah et al., 2024). Chronic sleep disturbance may be associated with sustained psychological resource loss, which may make it more difficult for nurses to maintain well-being (Li et al., 2025b). From the perspective of conservation of resources theory, individuals strive to acquire, retain, and protect their valuable resources (Hobfoll et al., 2018; Tomaso et al., 2021). Consequently, the impact of sleep quality on general well-being may operate via complex pathways rather than a simple direct link. Identifying protective resources that may buffer the association between sleep quality and reduced general well-being is important for supporting nurses’ mental health.

Based on the conservation of resources theory, when continuous resource depletion occurs, individuals tend to utilize internal resources, ranging from psychological recovery to specific coping mechanisms, to safeguard their mental health (Hobfoll, 1989; Li et al., 2025b). Previous studies among college students have shown that psychological resilience may mediate the association between sleep quality and general well-being (Su and He, 2023). Psychological resilience is commonly defined as an individual’s ability to maintain or recover mental health in the face of adversity and may function as a protective resource (Arora et al., 2022). In contrast, clinical nurses frequently endure severe occupational stress and chronic sleep deprivation, making them particularly vulnerable to resource depletion and mental health decline (Zhang et al., 2024). Furthermore, adversity quotient reflects individuals’ cognitive appraisal and coping capacity and has been closely associated with psychological resilience and general well-being among clinical nurses (Luo et al., 2025; Peng et al., 2025). However, the roles these resources play in the association between sleep quality and general well-being among clinical nurses remain largely unexplored.

Therefore, grounded in the conservation of resources theory, this study aimed to achieve three specific objectives: (1) to examine the associations among sleep quality, psychological resilience, adversity quotient, and general well-being among nurses, (2) to test whether psychological resilience and adversity quotient independently mediated the association between sleep quality and general well-being, and (3) to examine whether psychological resilience and adversity quotient formed a serial mediating effect linking sleep quality to general well-being. Clarifying these associations may help identify modifiable protective resources for supporting nurses’ well-being in the context of persistent sleep-related challenges.

## Theoretical framework

Grounded in the conservation of resources theory, which posits that individuals are motivated to obtain, retain, and protect resources (Hobfoll, 1989), this study views general well-being as a positive psychological resource (Diener and Fujita, 1995; Hobfoll et al., 2018). Poor sleep quality is associated with disrupted restoration and sustained resource depletion (Tucker et al., 2008; Palmer et al., 2024). When one resource is depleted, other adequate resources may help buffer the impact of loss (Hobfoll et al., 2018). Psychological resilience and adversity quotient are personal resources that may help protect against psychological resource loss when available to a sufficient degree (Hobfoll et al., 2018). Furthermore, personal resources are not mobilized simultaneously; rather, as a foundational psychological capacity, psychological resilience may precede and further reinforce individuals’ cognitive and coping capacities (Chen et al., 2015). Based on this theory, the following hypotheses were proposed (Figure 1).

**FIGURE 1:**
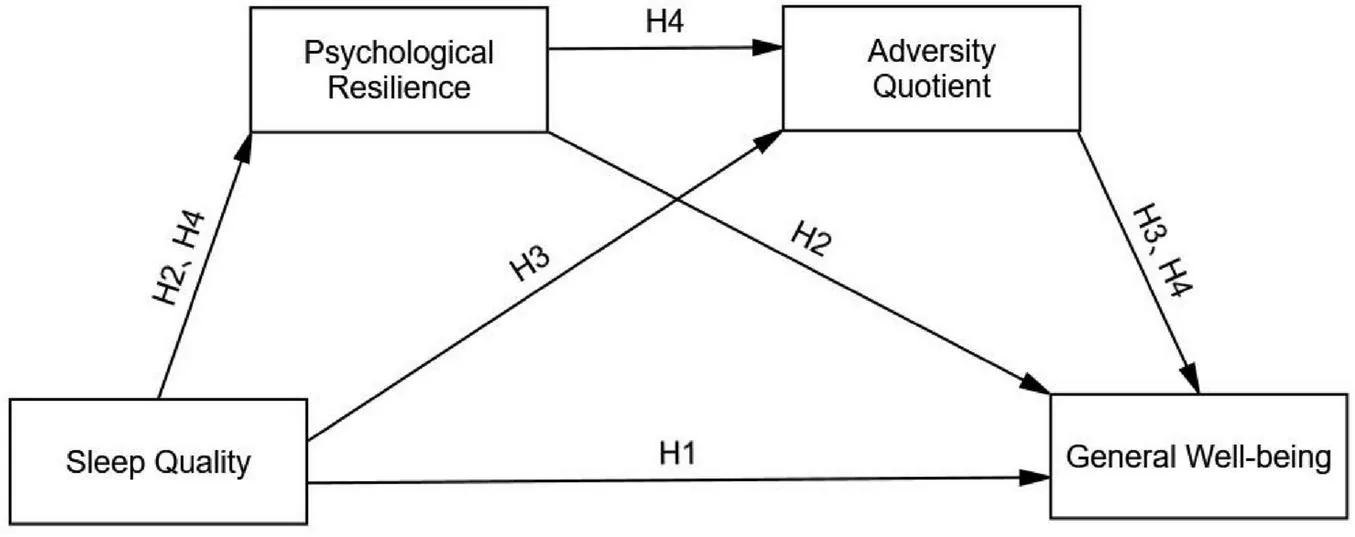
Conceptual framework and hypothesis.

H1: Poorer sleep quality would be associated with lower general well-being among nurses.

H2: Psychological resilience would mediate the association between sleep quality and general well-being.

H3: Adversity quotient would mediate the association between sleep quality and general well-being.

H4: Psychological resilience and adversity quotient would form a serial mediating effect between sleep quality and general well-being.

## Methods

### Study design

A multicenter cross-sectional study was conducted among nurses in Zhejiang Province, China, from September to November 2025. The study was reported in accordance with the STROBE guidelines for cross-sectional studies (von Elm et al., 2008).

### Sampling procedure

A multistage sampling approach was employed. In the first stage, six cities in Zhejiang Province were randomly selected. In the second stage, two to four tertiary hospitals were randomly selected from each sampled city, yielding 22 hospitals in total. In the third stage, after institutional approval was obtained from hospital administrators, nursing managers and trained research assistants jointly recruited nurses from each hospital using convenience sampling, with approximately 50 nurses enrolled per hospital on average. To enhance data quality, anonymous questionnaires were completed independently by participants, while trained research staff conducted face-to-face surveys at designated recruitment sites in each hospital and provided guidance during questionnaire completion.

### Participants and sample size

The inclusion criteria were: (1) registered nurses with valid nursing qualification certificates; (2) nurses who had worked in clinical nursing for at least one year; and (3) nurses who provided informed consent and participated voluntarily. The exclusion criteria were: (1) non-frontline clinical nurses; and (2) nurses who had experienced major life events that could affect their emotional state within the previous three months. Based on the guideline that the sample size should be 10 to 20 times the number of independent variables (n = 29), the recommended sample size ranged from 290 to 580 (Sun and Xu, 2014). To account for invalid questionnaires and participant dropouts, the target sample size was increased by approximately 20%, resulting in a range of 348 to 696. Questionnaires were checked for completeness, and 59 invalid questionnaires were excluded. Finally, 1,149 valid questionnaires were retained, yielding an effective response rate of 95.1%.

### Measurement instruments

#### General information questionnaire

A self-designed questionnaire was used to collect socio-demographic characteristics, including age (20–29, 30–39, and ≥ 40), sex (male and female), marital status (unmarried, married, and divorced or widowed), education (junior college, undergraduate, and postgraduate), department (medical department, surgical department, operating room, emergency department, intensive care unit, and others), years of professional experience (<3, 3–5, 5–10, and >10), professional title (senior nurse, charge nurse, associate superintendent nurse, and superintendent nurse), employment relationship (labor dispatch employees, civil servant status, official hospital staffing, and others), and whether to work the night shift or not.

#### Pittsburgh sleep quality index (PSQI)

The Pittsburgh Sleep Quality Index was developed by Buysse et al. at the University of Pittsburgh in 1989. It is mainly used to assess individuals’ subjective sleep quality during the past month (Buysse et al., 1989). The scale was later translated and adapted into Chinese by Liu et al. and has shown good reliability and validity in Chinese populations (Liu et al., 1996). The scale includes seven dimensions: subjective sleep quality, sleep latency, sleep duration, habitual sleep efficiency, sleep disturbances, use of sleep medication, and daytime dysfunction. Each dimension is scored from 0 to 3, with a total score ranging from 0 to 21. Higher scores indicate poorer sleep quality. In this study, Cronbach’s α for the scale was 0.873.

#### Adversity quotient scale (AQ)

The Adversity Quotient Scale, developed by Stoltz, was used to assess individuals’ adversity coping capacity (Stoltz, 1999). The Chinese revised version of this scale, developed by Liu et al., was applied to measure nurses’ adversity coping capacity (Liu et al., 2022). The scale contains 44 items across four dimensions: control, ownership, reach, and endurance. Items are rated on a 5-point Likert scale. Higher scores indicate stronger adversity response capacity. In this study, Cronbach’s α for the scale was 0.969.

#### Connor-Davidson resilience scale (CD-RISC)

The Connor-Davidson Resilience Scale, originally developed by Connor et al. and revised into a Chinese version by Yu et al., was used to assess individuals’ psychological resilience (Connor and Davidson, 2003; Yu and Zhang, 2007). The scale contains 25 items across three dimensions: strength, resilience, and optimism. Each item is rated on a 5-point Likert scale from 0 to 4. Higher scores indicate higher psychological resilience. In this study, Cronbach’s α for the scale was 0.961.

#### General well-being schedule (GWB)

The General Well-Being Schedule, originally developed by Fazio and revised into a Chinese version by Duan, was used to assess individuals’ general well-being (Fazio, 1977; Duan, 1996). The revised scale includes 18 items across six dimensions: satisfaction and interest in life, health concerns, energy, depressed or cheerful mood, emotional and behavioral control, and tension. Items 1, 3, 4, and 8 to 14 are scored from 1 to 6, items 2 and 5 are scored from 1 to 5, and items 15 to 18 are scored using a 0 to 10 visual analogue scale. Items 1, 3, 6, 7, 9, 11, 13, 15, and 16 are reverse-scored. Higher total scores indicate greater well-being. In this study, Cronbach’s α for the scale was 0.805.

### Statistical analyses

Data were analyzed using SPSS version 27.0 (IBM Corp., Armonk, NY, USA) and AMOS version 29.0 (IBM Corp., Armonk, NY, USA). Statistical significance was set at *P* < 0.05. Descriptive statistics were used to summarize participant characteristics. Continuous variables are presented as mean and standard deviation (SD), and categorical variables are presented as frequencies and percentages. Normality was assessed using skewness and kurtosis (Li, 2016). Pearson bivariate correlation coefficients were computed to examine associations among the study variables. Multicollinearity was assessed using tolerance values > 0.10 and variance inflation factor (VIF) values < 5.00 (Marquardt, 1970). Common method bias was evaluated using Harman’s single-factor test, with the first factor explaining less than 40% of the total variance as the conventional criterion (Zhou and Long, 2004). A common latent factor approach was also used to further assess whether common method bias substantially inflated the structural relationships among the variables (Podsakoff et al., 2012).

The measurement model was tested using confirmatory factor analysis (CFA). Model fit was evaluated using the Chi-square/degrees of freedom ratio (χ^2^/df), comparative fit index (CFI), Tucker-Lewis index (TLI), standardized root mean square residual (SRMR), and root mean square error of approximation (RMSEA). Values of χ^2^/df < 5, CFI > 0.90, TLI > 0.90, SRMR < 0.08, and RMSEA < 0.08 indicated acceptable fit (Browne and Cudeck, 1993). Convergent validity was assessed using standardized factor loadings, composite reliability, and average variance extracted (AVE). Discriminant validity was evaluated using the Fornell-Larcker criterion (Bagozzi and Yi, 1988).

The hypothesized model was tested using structural equation modeling (SEM). Sleep quality was specified as the independent variable, psychological resilience and adversity quotient as serial mediators, and general well-being as the outcome variable. Socio-demographic characteristics showing significant differences in the univariate analysis were included as covariates to control for their associations with the mediators and outcome variable, while preserving model parsimony. Among these covariates, categorical variables were dummy-coded, and binary variables were entered directly as dichotomous covariates. The indirect effect was tested using bias-corrected bootstrapping with 5,000 resamples. It was considered statistically significant when its 95% confidence interval did not include zero (Preacher and Hayes, 2008). Finally, the competing model was compared with the hypothesized model using χ^2^/df, CFI, TLI, SRMR, RMSEA, AIC, and BIC, with lower AIC and BIC values indicating better model fit (Burnham and Anderson, 2004).

## Results

### Sample characteristics

A total of 1,149 nurses were included in the final analysis. The sample was predominantly female (93.2%), aged 30–39 years (48.6%), and held an undergraduate degree (89.2%). More than half of the participants had over 10 years of professional experience (55.4%), and the largest professional-title group comprised senior nurses (62.0%). Most participants reported civil servant employment status (70.1%) and night-shift work (80.8%). Detailed demographic and occupational characteristics are presented in Table 1.

**TABLE 1:** Demographic characteristics of the participants (N = 1149).

Variables	N (%)	General well-being	U/H	*P*-value
Age			6.633	0.036
20–29	332 (28.9)	76.73 ± 0.69		
30–39	558 (48.6)	75.01 ± 0.53		
≥40	259 (22.5)	75.12 ± 0.77		
Sex			−0.258	0.796
Male	78 (6.8)	75.77 ± 1.50		
Female	1071 (93.2)	75.51 ± 0.38		
Education			5.412	0.067
Junior College	79 (6.9)	76.80 ± 1.42		
Undergraduate	1025 (89.2)	75.27 ± 0.39		
Postgraduate	45 (3.9)	79.18 ± 1.66		
Marital status			1.600	0.449
Unmarried	371 (32.3)	76.07 ± 0.68		
Married	757 (65.9)	75.37 ± 0.44		
Divorced or widowed	21 (1.8)	71.90 ± 3.24		
Years of professional experience			32.099	<0.001
<3	139 (12.1)	80.40 ± 1.07		
3–5	137 (11.9)	75.26 ± 1.04		
5–10	236 (20.5)	73.88 ± 0.84		
>10	637 (55.4)	75.14 ± 0.49		
Professional title			26.786	<0.001
Senior nurse	712 (62.0)	75.96 ± 0.48		
Charge nurse	370 (32.2)	74.05 ± 0.64		
Associate superintendent nurse	55 (4.8)	78.11 ± 1.50		
Superintendent nurse	12 (1.0)	83.92 ± 3.24		
Department			7.085	0.131
Medical department	460.00 (40.0)	75.72 ± 0.59		
Surgical department	91 (7.9)	75.77 ± 1.23		
Operating room	100 (8.7)	77.21 ± 1.07		
Emergency department	146 (12.7)	74.23 ± 1.10		
Intensive care unit	225 (19.6)	74.02 ± 0.90		
Others	127 (11.1)	77.54 ± 1.05		
Employment relationship			15.976	0.001
Labor dispatch employees	76 (6.7)	79.13 ± 1.47		
Civil servant status	805 (70.1)	74.65 ± 0.44		
Official hospital staffing	173 (15.1)	76.76 ± 0.95		
Others	95 (8.3)	77.93 ± 1.35		
Whether to work the night shift or not			−3.376	<0.001
Yes	928 (80.8)	74.94 ± 0.42		
No	221 (19.2)	78.03 ± 0.75		

### Descriptive statistics and univariate analyses

As shown in Table 2, the mean scores were 7.99 ± 3.66 for PSQI, 55.52 ± 14.91 for psychological resilience, 127.13 ± 31.38 for adversity quotient, and 75.53 ± 12.57 for general well-being. The absolute skewness and kurtosis values were below 2, indicating acceptable distributional characteristics. Univariate analyses showed significant differences in general well-being according to age, years of professional experience, professional title, employment relationship, and night-shift status.

**TABLE 2:** Descriptive statistics of study variables (N = 1149).

Variables	Score (Mean ± SD)
Pittsburgh Sleep Quality Index	7.99 ± 3.66
Subjective sleep quality	1.30 ± 0.81
Sleep latency	1.24 ± 0.75
Sleep duration	0.86 ± 0.97
Habitual sleep efficiency	1.10 ± 0.73
Sleep disturbances	0.40 ± 0.81
Use of sleep medication	1.46 ± 0.53
Daytime dysfunction	1.24 ± 0.75
General Well-being	75.53 ± 12.57
Satisfaction with life and interest in life	6.40 ± 1.91
Worry about health	7.05 ± 2.36
Energy	17.39 ± 4.36
Depressed or happy mood	15.23 ± 3.68
Control over emotions and behavior	12.66 ± 2.52
Relaxation and tension	16.80 ± 3.47
Psychological Resilience	55.52 ± 14.91
Strength	19.05 ± 4.98
Resilience	27.26 ± 8.46
Optimism	9.21 ± 2.58
Adversity Quotient	127.13 ± 31.38
Control	35.69 ± 9.13
Ownership	23.50 ± 6.17
Reach	41.08 ± 10.47
Endurance	26.87 ± 8.56

### Correlation and multicollinearity assessment

As shown in Table 3, higher PSQI scores, reflecting poorer sleep quality, were negatively correlated with psychological resilience, adversity quotient, and general well-being. Psychological resilience was positively correlated with adversity quotient and general well-being, and adversity quotient was positively correlated with general well-being. All correlations were statistically significant (*P* < 0.001). For the study variables, tolerance values ranged from 0.68 to 0.88, and VIF values ranged from 1.14 to 1.47, indicating no serious multicollinearity.

**TABLE 3:** Results of Pearson correlation analysis among study variables (N = 1149).

Variables	Pittsburgh Sleep Quality Index	Psychological resilience	Adversity quotient	General well-being
Pittsburgh Sleep Quality Index	1	1	1	1
Psychological Resilience	−0.283**			
Adversity Quotient	−0.317**	0.513**		
General Well-being	−0.484**	0.573**	0.578**	

**P < 0.001 (two-tailed)

### Common method bias

The first factor in the unrotated single-factor analysis accounted for 36.8% of the total variance, below the conventional 40% criterion. After introducing a common latent method factor, the model fit remained comparable to that of the corresponding model without the method factor. Changes in the standardized structural coefficients ranged from 0.014 to 0.028, and all major structural paths remained significant. These findings suggest that common method bias did not substantially affect the observed structural associations.

### Measurement model

The measurement model demonstrated acceptable fit: χ^2^/df = 4.169, CFI = 0.925, TLI = 0.912, SRMR = 0.047, and RMSEA = 0.053. All observed variables demonstrated statistically significant factor loadings ranging from 0.571 to 0.950 and exceeded the commonly recommended threshold of 0.40 (Browne and Cudeck, 1993). Composite reliability values ranged from 0.864 to 0.932, and AVE values ranged from 0.520 to 0.775, supporting convergent validity. The square root of AVE for each construct was greater than the absolute values of the inter-construct correlations, supporting discriminant validity. Detailed results are provided in Supplementary Tables 1 and 2.

### Structural model: hypothesized model testing

The hypothesized model showed acceptable fit to the data: χ^2^/df = 3.812, CFI = 0.923, TLI = 0.910, SRMR = 0.042, and RMSEA = 0.049. Figure 2 presents the hypothesized structural model with standardized path coefficients. Higher PSQI scores, indicating poorer sleep quality, were associated with lower psychological resilience (β = −0.389), lower adversity quotient (β = -0.232), and lower general well-being (β = −0.361). Psychological resilience was positively associated with adversity quotient (β = 0.434) and general well-being (β = 0.352), while adversity quotient was positively associated with general well-being (β = 0.300). Detailed path coefficients for all study variables and covariates are provided in Supplementary Table 3. Compared with the hypothesized model, the competing model, which specified the reverse ordering of the two mediators, showed poorer fit (χ^2^/df = 4.973, CFI = 0.891, TLI = 0.873, SRMR = 0.076, RMSEA = 0.059), with higher AIC and BIC values. Specifically, the competing model exceeded the hypothesized model by 435.042 in AIC and 419.903 in BIC, indicating that the hypothesized model was better supported.

**FIGURE 2:**
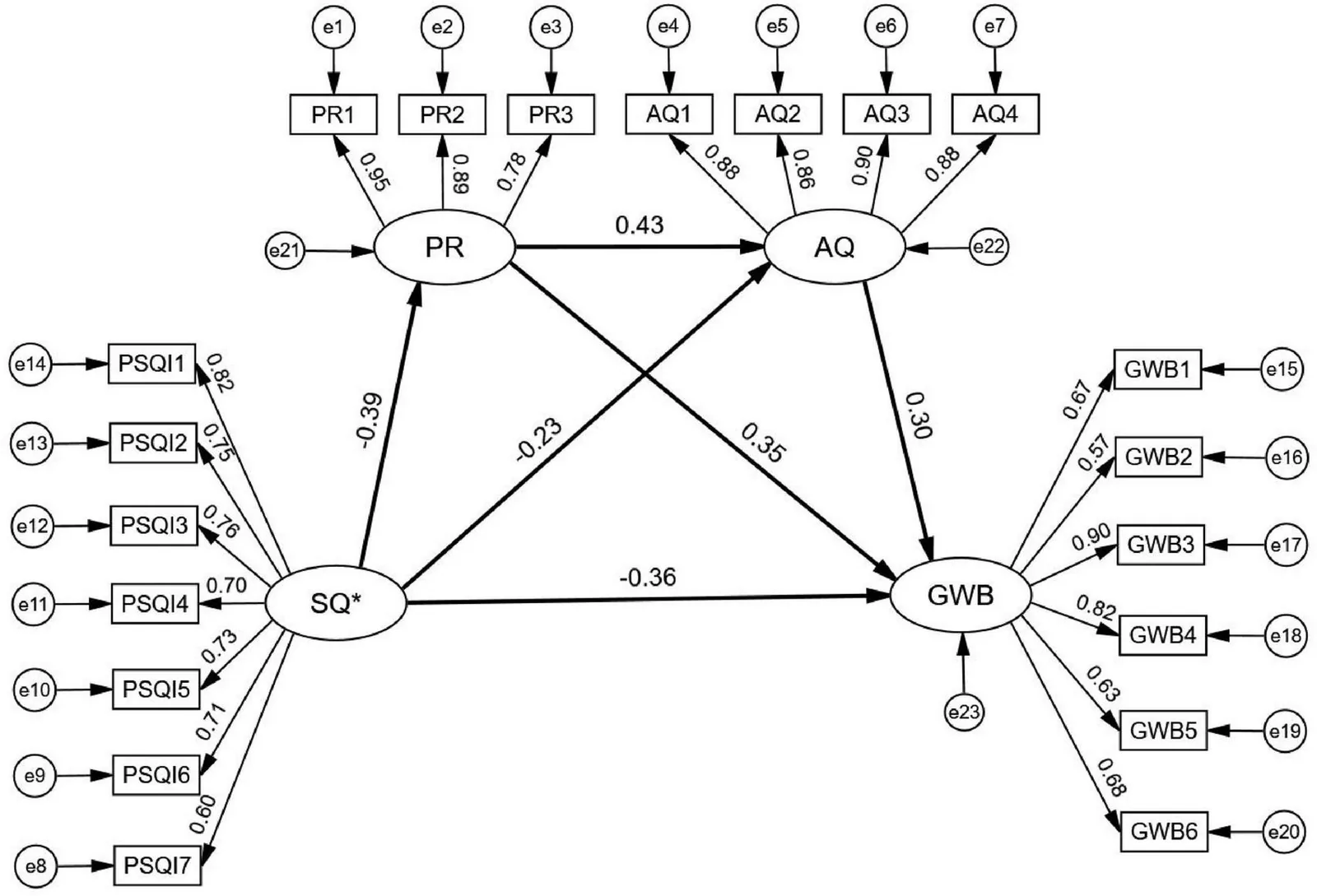
Mediating roles of psychological resilience and adversity quotient in the relationship between sleep quality and general well-being. Standardized coefficients (β) are shown. Covariates were included in the model but omitted from the figure for clarity. *SQ was assessed using the Pittsburgh Sleep Quality Index (PSQI), with higher PSQI scores indicating poorer sleep quality. SQ: sleep quality, PR: psychological resilience, AQ: adversity quotient, GWB: general well-being, PSQI1: subjective sleep quality, PSQI2: sleep latency, PSQI3: sleep duration, PSQI4: habitual sleep efficiency, PSQI5: sleep disturbances, PSQI6: use of sleep medication, PSQI7: daytime dysfunction, PR1: strength, PR2: resilience, PR3: optimism, AQ1: control, AQ2: ownership, AQ3: reach, AQ4: endurance, GWB1: satisfaction and interest in life, GWB2: health concern, GWB3: energy, GWB4: depressed or cheerful mood, GWB5: emotional and behavioral control, GWB6: tension.

### Direct and mediating effects

As shown in Table 4, the direct effect of sleep quality on general well-being was significant (β = −0.361, *P* < 0.001), accounting for 58.4% of the total effect. The total indirect effect was also significant (β = −0.257, *P* < 0.001), accounting for 41.6% of the total effect. Three specific indirect pathways were significant: Path 1: sleep quality → psychological resilience → general well-being (β = −0.137, *P* < 0.001). Path 2: sleep quality → adversity quotient → general well-being (β = −0.070, *P* < 0.001). Path 3: sleep quality → psychological resilience → adversity quotient → general well-being was significant (β = −0.051, *P* < 0.001).

**TABLE 4:** Direct, indirect, and total effects of the study variables.

Effects	Path relationship	B	Bootstrap SE	β	Bootstrap 95% CI	*P*-value	Proportion of total effect (%)*
Direct effect	Sleep quality → general well-being	−0.612	0.060	−0.361	(−0.725, −0.495)	<0.001	58.4
Indirect effect	Sleep quality → psychological resilience → general well-being	−0.232	0.031	−0.137	(−0.296, −0.173)	<0.001	22.2
	Sleep quality → adversity quotient → general well-being	−0.118	0.025	−0.070	(−0.174, −0.073)	<0.001	11.2
	Sleep quality → psychological resilience → adversity quotient → general well-being	−0.086	0.018	−0.051	(−0.124, −0.055)	<0.001	8.2
Total indirect effect		−0.436	0.046	−0.257	(−0.524, −0.345)	<0.001	41.6
Total effect		−1.049	0.074	−0.618	(−1.189, −0.904)	<0.001	100.0

*Proportion calculated based on the standardized coefficients (β).

## Discussion

Guided by conservation of resources theory, this study examined the relationship between sleep quality and general well-being among nurses, and further explored the sequential mediating roles of psychological resilience and adversity quotient. Three principal findings emerged. First, poorer sleep quality was significantly associated with lower general well-being. Second, psychological resilience and adversity quotient each showed significant independent indirect associations in this relationship. Third, a significant hypothesized serial pathway involving sleep quality, psychological resilience, adversity quotient, and general well-being was supported. Collectively, the indirect associations accounted for 41.6% of the total association. These findings reveal a resource-related pattern linking sleep quality to nurses’ general well-being through sequential personal resources, providing actionable insights for nursing managers to develop targeted strategies for promoting nurses’ well-being.

The structural equation model showed relatively strong explanatory power for the serial indirect association of psychological resilience and adversity quotient in the relationship between sleep quality and general well-being among nurses. Compared with a conventional mediation model, the present model can represent abstract constructs such as general well-being and psychological resilience as latent variables, using scale dimensions as observed indicators (Anderson and Gerbing, 1988). This approach is more conducive to controlling measurement error and provides a more accurate estimate of the indirect effects of psychological resilience and adversity quotient (Gerbing and Anderson, 1985). Moreover, compared with the reverse-order competing model, the proposed serial mediation model fit the data better, suggesting that the sequence from psychological resilience to adversity quotient may be more consistent with the observed pattern in this study. Nevertheless, these findings were derived from cross-sectional data and model comparison, and therefore do not imply causality. Future longitudinal or intervention studies should test whether this sequence holds across time and setting.

This study revealed a moderate level of general well-being among nurses, which is broadly consistent with previous studies conducted in China (Fan et al., 2024). Compared with earlier research, the overall level of general well-being among Chinese nurses has demonstrated an upward trend (Sun et al., 2013). This upward trend may be attributed to recent structural initiatives within China’s healthcare system, including the comprehensive nationwide expansion of high-quality nursing services, the optimization of nurse staffing and shift-scheduling standards, and enhanced policy protections for clinical nurses’ occupational safety and compensation incentives (Wu, 2023; Du et al., 2025). However, the occupational demands of clinical nursing remain high, and nurses’ daily recovery faces persistent challenges, leaving their general well-being vulnerable to erosion (Hernandez et al., 2024). Maintaining psychological functions, such as emotional regulation and stress coping, relies on adequate physiological restoration, which is impaired by poor sleep quality (Vandekerckhove and Wang, 2017). As a vital restorative process, sleep quality is essential for maintaining psychological reserves and daily coping capacity. This study revealed a positive association between sleep quality and general well-being among nurses, which is consistent with previous findings in the literature (Zhang et al., 2025). Moreover, suboptimal sleep quality remains a prevalent issue among clinical nurses (Zhang et al., 2023). Although improving sleep quality is crucial for safeguarding nurses’ well-being, achieving sustained changes remains challenging in practice due to persistent staffing constraints and the demanding nature of clinical workflows (Li et al., 2025a).

Psychological resilience showed a significant independent indirect association in the relationship between sleep quality and general well-being among nurses. This finding is plausible because psychological resilience, as an important internal resource, depends partly on adequate sleep for its maintenance and recovery, and may reinforce one another over time in high-stress healthcare settings (Arora et al., 2022; Wu et al., 2024). When sleep quality is compromised, nurses working under heavy clinical demands may experience insufficient recovery and greater resource depletion, which may be associated with lower psychological resilience. As a vital self-regulatory buffer against occupational strain, lower psychological resilience may make it more difficult for nurses to maintain general well-being (Cooper et al., 2021). However, compared with findings from studies of student populations, the mediating effect of psychological resilience was relatively modest among nurses (Su and He, 2023). This difference may stem from the distinct structural nature of their environments and resource-drain patterns. While students often experience more episodic and temporary sleep insufficiency, clinical nurses are frequently embedded in sustained occupational strain, where sleep disturbances and heavy workloads intertwine (Sun et al., 2019; Wu et al., 2024). Under such chronic systemic pressure, resource depletion occurs across multiple physiological and psychological domains simultaneously (Hobfoll et al., 2018). Therefore, the explanatory contribution of psychological resilience as a single indirect association may be limited.

Adversity quotient also showed a significant independent indirect association in the relationship between sleep quality and general well-being. The results showed that nurses with poorer sleep quality tended to have a lower adversity quotient, which was in turn associated with lower general well-being. As a coping-related personal resource, adversity quotient reflects individuals’ cognitive appraisal and outward behavioral coping capacity when facing stress or adversity (Luo et al., 2025). Impaired sleep quality may interfere with the recovery of cognitive and emotional functioning and may therefore be associated with lower adversity quotient (Palmer et al., 2024). Previous studies have shown that individuals with lower adversity quotient may have greater difficulty using effective coping strategies and may be more vulnerable to negative stress appraisals (Gou et al., 2024). This could be linked to further psychological resource loss and lower general well-being (Hobfoll et al., 2018). However, the mediating effect of adversity quotient was smaller than that of psychological resilience. Drawing on conservation of resources theory, psychological resilience may reflect a foundational internal reserve associated with general physiological and emotional recovery, aligning with its role as a primary correlate of sleep-related well-being (Cooper et al., 2020). By contrast, adversity quotient captures a more specific, action-oriented capacity linked to navigating acute workplace stressors; its situational focus yields a smaller indirect effect than the systemic buffering of resilience (Hobfoll et al., 2018).

A key contribution of this study was the support for a significant hypothesized serial pathway linking sleep quality, psychological resilience, adversity quotient, and general well-being. Although the serial indirect association was modest in magnitude, it remained statistically significant, suggesting that sleep quality, psychological resilience, adversity quotient, and general well-being may be interrelated rather than operating as isolated factors. From the perspective of conservation of resources theory, sleep deprivation impairs physiological recovery and emotional regulation (Troy et al., 2023). Such regulatory capacity is closely related to psychological resilience (Xu et al., 2026). Under conditions of chronic strain, individuals may lack sufficient emotional and cognitive resources to continuously manage external demands, rendering adaptive coping through adversity quotient even more difficult (Xiao et al., 2026). Even modest indirect associations may be meaningful in complex clinical settings, where nurses’ general well-being is closely associated with multiple interacting factors (Xiao et al., 2022; Xu et al., 2026). Repeated day-to-day sleep disturbances may be associated with poorer psychological functioning and well-being over time (Gifkins et al., 2020).

## Practical implications of the findings

This study offers several practical implications for improving nurses’ general well-being, which should be interpreted with caution given the cross-sectional design. First, sleep quality should be prioritized as the primary target for intervention, because it was directly associated with general well-being and may also shape nurses’ personal resources. Nursing managers may consider optimizing shift schedules, reducing consecutive night shifts, protecting rest time between shifts, and strengthening fatigue management and sleep health education. Second, interventions should aim to preserve and enhance psychological resilience, for example through structured resilience training, stress-management programs, and timely psychological support. Finally, adversity quotient should also be strengthened by helping nurses develop more effective coping strategies and better adaptive responses to high-demand clinical work. Overall, a sleep-centered and resource-oriented approach may be more effective in supporting nurses’ general well-being than focusing on any single factor alone.

## Limitations

Several limitations should be acknowledged in this study. First, the cross-sectional design limits causal inference and temporal interpretation of the observed associations. Longitudinal or intervention studies are needed to further examine the directionality of relationships among sleep quality, psychological resilience, adversity quotient, and general well-being. Second, research variables were assessed using self-reported measures, which may be subject to response bias. Although common method bias was examined, it cannot be fully ruled out. Third, although relevant covariates were controlled, other occupational and psychosocial factors, such as workload intensity and organizational support, were not fully examined and should be considered in future research. Fourth, participants were recruited from tertiary medical institutions in Zhejiang Province, which may limit the generalizability of the findings to nurses in other regions, hospital levels, and healthcare systems.

## Conclusion

This study drew on conservation of resources theory to examine the association between sleep quality and general well-being among nurses and to test the mediating roles of psychological resilience and adversity quotient in this association. The findings showed that nurses reported a moderate level of general well-being. Poorer sleep quality was not only directly associated with lower general well-being, but also indirectly associated with it through lower psychological resilience and adversity quotient as separate mediators, as well as through the serial mediation of psychological resilience and adversity quotient. This study extends current understanding of the association between sleep quality and general well-being among nurses and provides a resource-oriented perspective for nursing management. Based on these findings, nursing administrators may consider prioritizing sleep management and strengthening psychological resilience and adversity-related coping resources when developing strategies to support nurses’ general well-being.

## Data Availability

The raw data supporting the conclusions of this article will be made available by the authors, without undue reservation.
