# Supplementary material for: Associations between sleep quality and well-being among nurses: the serial mediating roles of psychological resilience and adversity coping
Source: Front Psychol. 2026 Sep 30;17:1962283. doi: 10.3389/fpsyg.2026.1962283 (PMC13661319; doi:10.3389/fpsyg.2026.1962283)
Supplement: Supplementary file 1 [file Data_Sheet_1.pdf]

Supplementary Table 1. Factor loadings and convergent validity indices for the measurement model

**Variable**	**Factor**	**Factor loading**				**CR**	**AVE**
		**B**	**S.E.**	***β***	**P**		
Pittsburgh Sleep Quality Index	Subjective sleep quality	0.667	0.021	0.821	< 0.001	0.886	0.529
	Sleep latency	0.507	0.025	0.750	< 0.001		
	Sleep duration	0.622	0.022	0.761	< 0.001		
	Habitual sleep efficiency	0.392	0.030	0.704	< 0.001		
	Sleep disturbances	0.533	0.020	0.728	< 0.001		
	Use of sleep medication	0.442	0.024	0.710	< 0.001		
	Daytime dysfunction	0.436	0.017	0.596	< 0.001		
Psychological Resilience	Strength	4.728	0.113	0.950	< 0.001	0.906	0.763
	Resilience	7.484	0.201	0.885	< 0.001		
	Optimism	2.003	0.065	0.776	< 0.001		
Adversity Quotient	Control	8.074	0.215	0.884	< 0.001	0.932	0.775
	Ownership	5.271	0.148	0.855	< 0.001		
	Reach	9.464	0.243	0.904	< 0.001		
	Endurance	7.502	0.202	0.877	< 0.001		
General Well-being	Satisfaction and interest in life	1.286	0.052	0.673	< 0.001	0.864	0.520
	Health concern	1.144	0.073	0.571	< 0.001		
	Energy	3.920	0.104	0.900	< 0.001		
	Depressed or cheerful mood	3.014	0.092	0.820	< 0.001		
	Emotional and behavioral control	1.594	0.070	0.632	< 0.001		
	Tension	1.662	0.102	0.679	< 0.001		

Supplementary Table 2. Correlations among study variables and discriminant validity based on the Fornell-Larcker criterion.

**Variable**	**Pittsburgh Sleep Quality Index**	**Psychological Resilience**	**Adversity Quotient**	**General Well-being**
Pittsburgh Sleep Quality Index	**0.727**			
Psychological Resilience	-0.385	**0.874**		
Adversity Quotient	-0.424	0.543	**0.880**	
General Well-being	-0.620	0.657	0.642	**0.721**

Note: Diagonal values are square roots of average variance extracted (AVE) and other values are latent correlations.

Supplementary Table 3. Path coefficients of the structural equation model linking sleep quality, psychological resilience, adversity quotient, and general well-being after controlling for covariates.

**Investigation Content**	**Outcome Variables**														
	**Psychological Resilience**					**Adversity Quotient**					**General Well-being**				
	**B**	**SE**	**C.R**	***β***	***P***	**B**	**SE**	**C.R.**	***β***	***P***	**B**	**SE**	**C.R.**	***β***	***P***
**Age (ref: 20-29)**															
30-39	0.062	0.127	0.486	0.028	0.627	-0.148	0.123	-1.203	-0.059	0.229	0.298	0.158	1.885	0.088	0.059
> 40	0.134	0.165	0.814	0.050	0.416	-0.356	0.157	-2.263	-0.119	0.024	0.389	0.195	1.998	0.096	0.046
**Years of professional experience (ref: < 3)**															
3-5	-0.318	0.134	-2.384	-0.093	0.017	-0.320	0.136	-2.352	-0.083	0.019	0.018	0.150	0.123	0.004	0.902
5-10	-0.412	0.143	-2.874	-0.150	0.004	-0.413	0.146	-2.832	-0.133	0.005	-0.007	0.185	-0.040	-0.002	0.968
> 10	-0.362	0.170	-2.132	-0.162	0.033	-0.268	0.163	-1.637	-0.106	0.102	-0.084	0.210	-0.400	-0.025	0.689
**Professional title (ref: Senior nurse)**															
Charge nurse	0.166	0.085	1.941	0.070	0.052	0.052	0.082	0.633	0.019	0.527	-0.153	0.094	-1.632	-0.042	0.103
Associate superintendent nurse	0.607	0.167	3.623	0.117	< 0.001	0.171	0.156	1.098	0.029	0.272	0.159	0.186	0.859	0.020	0.390
Superintendent nurse	1.189	0.279	4.262	0.109	< 0.001	0.882	0.294	3.003	0.072	0.003	0.359	0.415	0.865	0.022	0.387
**Employment relationship (ref: Labor dispatch employees)**															
Civil servant status	-0.053	0.157	-0.341	-0.012	0.733	-0.033	0.134	-0.246	-0.007	0.806	0.128	0.149	0.861	0.019	0.389
Official hospital staffing	0.056	0.093	0.603	0.018	0.547	-0.035	0.096	-0.364	-0.010	0.716	0.106	0.113	0.939	0.022	0.348
Other	-0.130	0.133	-0.974	-0.032	0.330	0.109	0.145	0.753	0.024	0.452	0.206	0.157	1.310	0.033	0.190
**Whether to work the night shift or not**	-0.029	0.093	-0.314	-0.010	0.754	-0.203	0.098	-2.072	-0.064	0.038	0.091	0.098	0.926	0.021	0.354
**Pittsburgh sleep quality index**	-0.432	0.045	-9.668	-0.389	< 0.001	-0.290	0.051	-5.638	-0.232	< 0.001	-0.612	0.060	-10.238	-0.361	< 0.001
**Psychological resilience**						0.490	0.056	8.757	0.434	< 0.001	0.538	0.056	9.551	0.352	< 0.001
**Adversity quotient**											0.406	0.049	8.299	0.300	< 0.001
**R^2**	0.190					0.363					0.652				
